# Identification and immobilization of a novel cold-adapted esterase, and its potential for bioremediation of pyrethroid-contaminated vegetables

**DOI:** 10.1186/s12934-017-0767-9

**Published:** 2017-09-11

**Authors:** Xinjiong Fan, Weiqu Liang, Yanfang Li, He Li, Xiaolong Liu

**Affiliations:** 1 School of Basic Medical Sciences, Anhui Medical University, 81 Meishan Rd, Hefei, 230032 Anhui People’s Republic of China; 2 Dongguan Agriculture Research Center, Dongguan, 523079 Guangdong People’s Republic of China; 3 School of Basic Courses, Guangdong Pharmaceutical University, 280 E. Outer Ring Rd., Guangzhou, 510006 Guangdong People’s Republic of China

**Keywords:** Pyrethroid, Esterase, Immobilization, Reusability, Bioremediation, Vegetables

## Abstract

**Background:**

Pyrethroids are potentially harmful to living organisms and ecosystems. Thus, concerns have been raised about pyrethroid residues and their persistence in agricultural products. To date, although several pyrethroid-hydrolyzing enzymes have been cloned, very few reports are available on pyrethroid-hydrolyzing enzymes with cold adaptation, high hydrolytic activity and good reusability, indispensable properties in practical bioremediation of pyrethroid-contaminated vegetables.

**Results:**

Here, a novel gene (*est684*) encoding pyrethroid-hydrolyzing esterase was isolated from the Mao-tofu metagenome for the first time. Est684 encoded a protein of 227 amino acids and was expressed in *Escherichia coli* BL21 (DE3) in soluble form. The optimum temperature was 18 °C. It maintained 46.1% of activity at 0 °C and over 50% of its maximal activity at 4–35 °C. With the goal of enhancing stability and recycling biocatalysts, we used mesoporous silica SBA-15 as a nanometer carrier for the efficient immobilization of Est684 by the absorption method. The best conditions were an esterase-to-silica ratio of 0.96 mg/g (w/w) and an adsorption time of 30 min at 10 °C. Under these conditions, the recovery of enzyme activity was 81.3%. A large improvement in the thermostability of Est684 was achieved. The half-life (T_1/2_) of the immobilized enzyme at 35 °C was 6 h, 4 times longer than the soluble enzyme. Interestingly, the immobilized Est684 had less loss in enzyme activity up to 12 consecutive cycles, and it retained nearly 54% of its activity after 28 cycles, indicating excellent operational stability. Another noteworthy characteristic was its high catalytic activity. It efficiently hydrolyzed cyhalothrin, cypermethrin, and fenvalreate in pyrethroid-contaminated cucumber within 5 min, reaching over 85% degradation efficiency after four cycles.

**Conclusions:**

A novel cold-adapted pyrethroid-hydrolyzing esterase was screened from the Mao-tofu metagenome. This report is the first on immobilizing pyrethroid-hydrolyzing enzyme on mesoporous silica. The immobilized enzyme with high hydrolytic activity and outstanding reusability has a remarkable potential for bioremediation of pyrethroid-contaminated vegetables, and it is proposed as an industrial enzyme.

**Electronic supplementary material:**

The online version of this article (doi:10.1186/s12934-017-0767-9) contains supplementary material, which is available to authorized users.

## Background

Pyrethroids are one of the most important insecticides globally. They already have high usage, and the total demand continues to grow. Pyrethroid residues are toxic, which may presents a risk to human health and poses a significant threat to ecosystems [1, 2]. Enzyme biotechnology offers an attractive solution for the removal of highly toxic pollutants such as pyrethroid residues. The main metabolic pathway of pyrethroids includes oxidation and ester-bond hydrolysis, which produces nontoxic acid and alcohol compounds [3, 4]. Native enzymes, such as PytY, EstP, PytH, Pye3, PytZ, EstSt7, and CMO, have been explored for the biodegradation of pyrethroids in the past 10 years [5–10]. Very few reports are available on pyrethroid-hydrolyzing enzyme with novel characteristics, such as cold adaptation and high hydrolytic activity, because such enzymes are rare. Cold-adapted enzymes exhibit high catalytic efficiency at moderate and low temperatures and are thus versatile biocatalysts in many applications [11], especially for bioremediation of pyrethroid-contaminated vegetables.

However, the cost of the enzyme is a key determinant of the economic feasibility of biotransformations. The ability to fully recycle biocatalysts would help to minimize waste disposal and optimize economic benefits. Enzyme immobilization might be the best method for reducing the cost of the process because it would permit reuse of the enzymes [12]. Enzymes immobilized on the surface of functional materials have many advantages over soluble enzymes, including the possibility for recovery and reuse, enhanced stability and catalytic activity, and easy operation [13–16]. Several methods are available for immobilizing proteins onto solid supports, and they are divided into four main categories: adsorption, entrapment, cross-linking, and covalent binding using commercial materials [17]. Each method has advantages and disadvantages. In physical adsorption, enzyme conformation is largely protected because the adsorption is mainly achieved by either electrostatic interaction or van der Waals’ force [18]. However, such bonding is relatively weak, and enzyme leakage from carrier can occur during operational process. Sol–gel entrapment is widely used because of its mild reaction conditions. Various methods for sol–gel formation have been proposed for building nanostructures and immobilizing enzymes. However, some limitations exist, such as enzyme leakage from the support. Cross-linking and covalent binding have the advantage of strong irreversible binding of the enzyme to the supporting agent, which can protect against enzyme leakage. A drawback of this strong binding is the risk of a reduction in enzyme activity [19].

In addition to the different immobilization approaches, a variety of carriers have been used for enzyme immobilization. Mesoporous silica materials have attracted significant interest because of their stable structures, larger surface areas, good biocompatibility, and tunable pore sizes and volumes [20–22]. As immobilizing carriers, mesoporous materials can incorporate proteins or enzymes through physical or chemical reactions with good adsorption because of their large specific surface area. The available size range, about 3–10 nm, is comparable with the hydrodynamic radius of most enzymes being used. Although many reports exist on the properties of enzymes immobilized on mesoporous silica, the effect of mesoporous silica on the properties of pyrethroid-hydrolyzing esterase has not yet been investigated.

In this study, we reported the cloning, identification and biochemical characterization of one novel cold-adapted pyrethroid-hydrolyzing esterase derived from the Mao-tofu metagenome. With the goal of enhancing stability and recycling biocatalysts, we immobilized this esterase on a matrix of mesoporous silica SBA-15. The stability and reusability of the immobilized enzyme were estimated and compared with the soluble enzyme. In addition, the potential use of the immobilized pyrethroid-hydrolyzing esterase for removal of pyrethroids on vegetables was investigated.

## Results and discussion

### Screening for pyrethroid-hydrolyzing esterase from a metagenomic library

Mao-tofu, is a type of fermented tofu, is primarily fermented by *Mucor* spp., which can degrade the starches, proteins, and lipids of soybean [23]. Presumably, there are a variety of hydrolytic enzymes excreted by Mao-tofu microbes. The metagenomic library-based technique has been successfully applied to screen novel genes [24]. Here, the total DNA was extracted from Mao-tofu microbes and used to construct a metagenomic library containing approximately 13,200 clones. Several transformants with hydrolysis zones were obtained by functional expression screening of plasmid clones with esterase/lipase activity. One of them harboring the pUC118No.2 showed pyrethroid-hydrolyzing activity.

### Genetic characterization

**Fig. 1:**
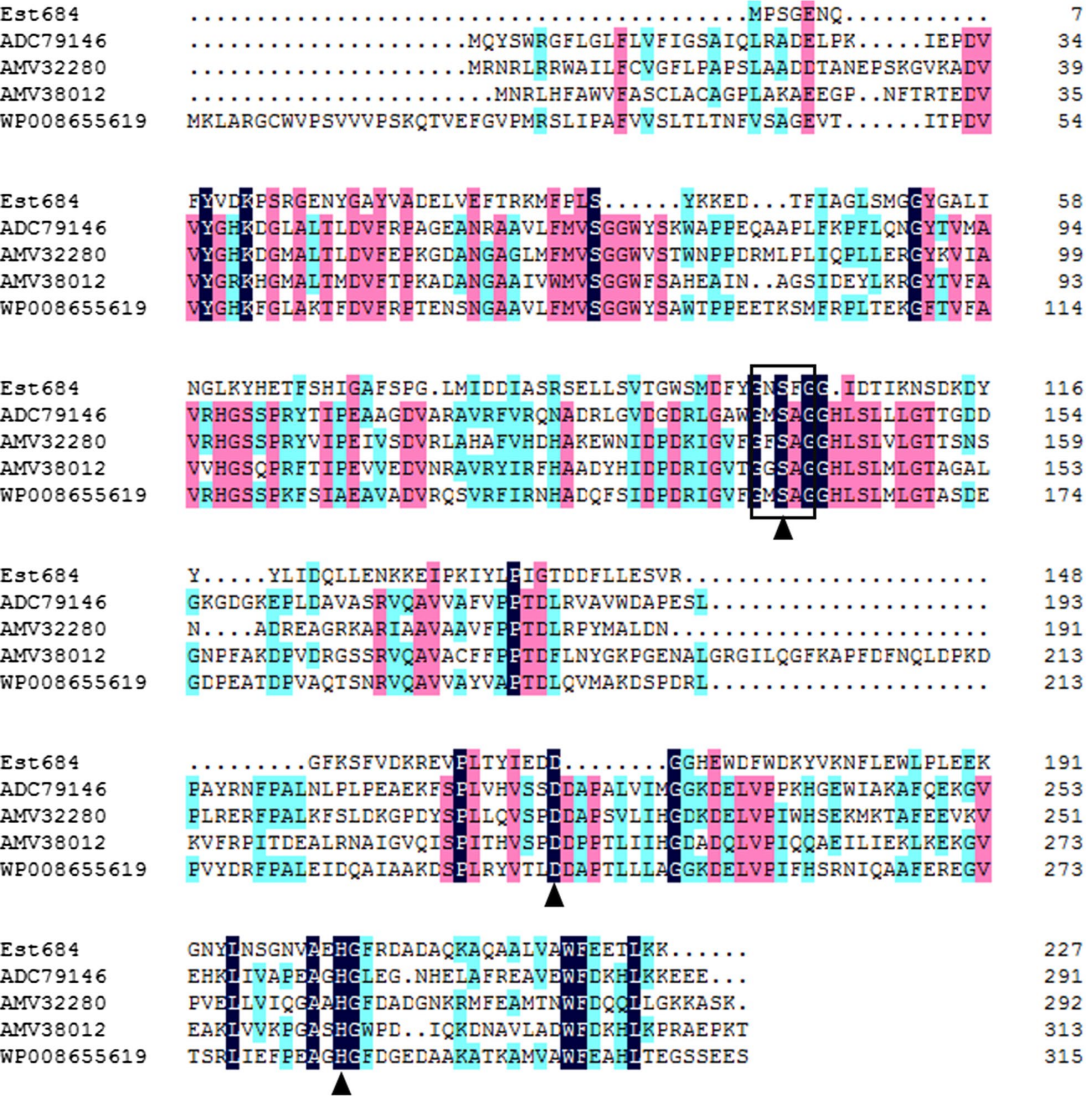
Multiple amino acid sequence alignment of Est684. Multiple alignment of the partial amino acid sequences containing the conserved motifs of G-X-S-X-G and putative catalytic triad residues of alpha/beta hydrolase family proteins. The protein sequences were retrieved from GenBank. The accession numbers of the aligned sequences are for the following organisms: ADC79146, lipase/esterase from uncultured sludge bacterium; AMV32280, acetylxylan esterase precursor from *Pirellula* sp. SH-Sr6A; AMV38012, acetylxylan esterase precursor from *Planctomyces* sp. SH-PL62; WP008655619, alpha/beta hydrolase from *Rhodopirellula europaea*. The alignment was carried out using the Clustal W method. The open boxes indicate amino acid residues belonging to the putative catalytic triad residues, and triangles denote the active site

Plasmid pUC118No.2 had an insert of 2336 bp. Based on the sequence analysis, the ORF that encoded a 227 amino acids protein was identified as a putative lipase/esterase gene (designated as *est684*) with a predicted molecular mass (Mr) of 24.97 kDa. The putative amino acid sequence of Est684 was used to perform a BLAST program provided by the NCBI and Swissprot databases. There was moderate identity between Est684 and other hydrolases. This encoded protein showed highest identity with alpha/beta hydrolase fold family protein from *Enterococcus silesiacus* strain LMG 23085 (497/614, 81% identity). Multiple sequence alignment of Est684 and lipolytic proteins revealed the typical catalytic triad of active site serine (S103) motif G-X-S-X-G (Fig. 1), conserved aspartic acid (D167), and histidine (H203) residue motif in the encoded protein [25], similar to most lipases/esterases [26]. This DNA sequence has been submitted to GenBank under Accession Number (MF437038).

### Heterologous expression and purification of recombinant Est684

Est684 was cloned and expressed in *Escherichia coli* BL21 (DE3), and then purified by a Ni–NTA His·Bind column and a Superdex 200 (16/60) size-exclusion column. SDS-PAGE analysis showed that Est684 migrates as a single band with a molecular mass lower than 35 kDa (Additional file 1: Figure S1), in accordance with its predicted molecular mass of 30.58 kDa, containing the 227 amino acids and a fusion of 51 amino acids corresponding to polyhistidine tag (His-tag), a unique thrombin cleavage site (Thrombin).

### Substrate specificity of Est684

**Fig. 2:**
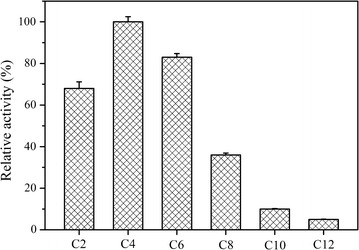
Substrate specificity of Est684 against various ρ-nitrophenyl esters with acyl chain lengths of C2, C4, C6, C8, C10, and C12. Data points are the average of triplicate measurements, and error bars represent the standard deviation

Est684 belongs to the alpha/beta hydrolase. For convenience, *p*-nitrophenyl esters, as general substrates of esterases/lipases, were chosen to measure substrate specificity. The catalytic reaction was tested at 18 °C and pH 6.5 for 5 min. Est684 displayed the highest activity towards *p*-nitrophenyl butyrate among all tested *p*-nitrophenyl esters (Fig. 2).

### Effect of temperature on the activity and stability of recombinant Est684

**Fig. 3:**
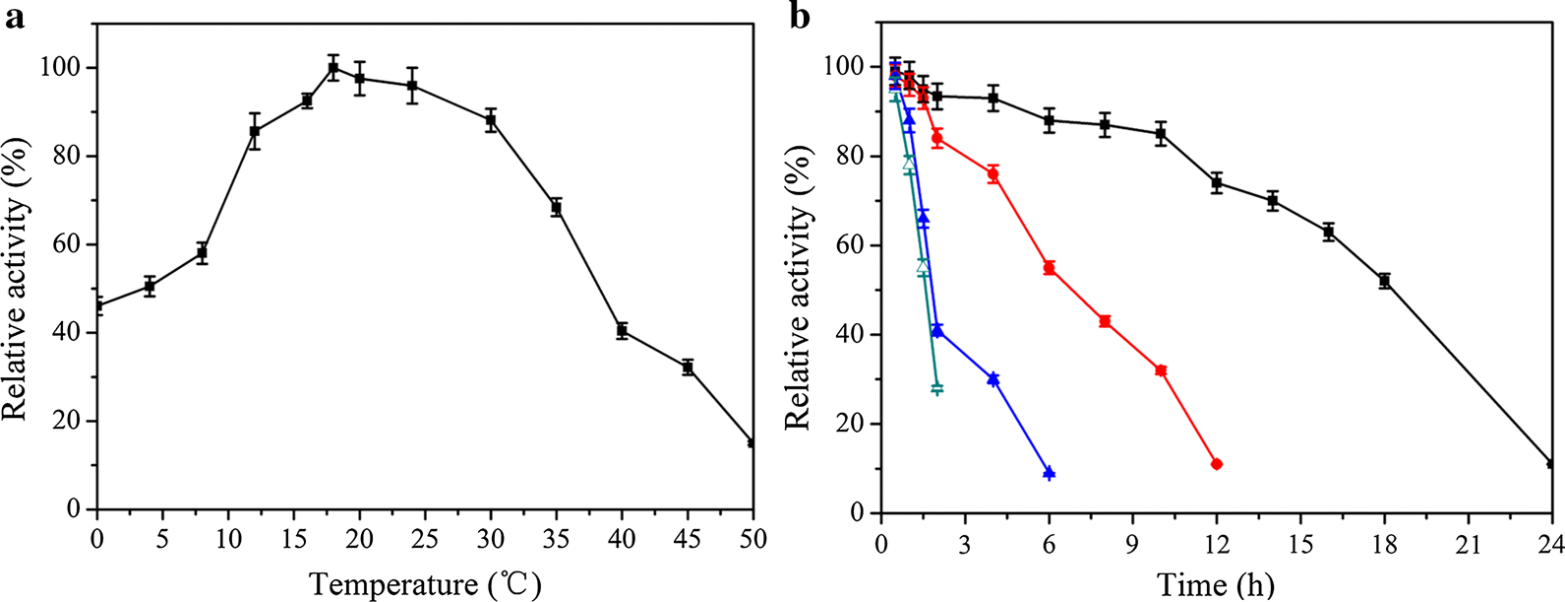
Effect of temperature on the activity and stability of Est684. **a** The optimum temperature was determined analogously by measuring esterase activity in the temperature range of 0–50 °C in a phosphate buffer (50 mM, pH 6.5). **b** Thermostability was measured by preincubation of enzymes in 50 mM potassium phosphate buffer (pH 6.5) at 18 (filled square), 25 (filled circle), 30 (filled triangle) and 35 °C (open triangle) for different intervals. Data points are the average of triplicate measurements, and error bars represent the standard deviation

To investigate the effect of temperature on the activity of Est684, the catalytic activity was measured at different temperatures ranging from 0 to 50 °C with *p*-nitrophenyl butyrate as a substrate, with the consideration of practical bioremediation of pyrethroid-contaminated agricultural products. The optimum of Est684 was 18 °C (Fig. 3a). It maintained 46.1% of the maximum activity at 0 °C, and more than 85% at 12 °C. This remarkable activity at low temperatures indicates that Est684 was a cold-adapted enzyme. Moreover, it maintained more than 50% of its maximal activity at 4–35 °C, indicating that Est684 possessed good adaptability at moderate and low temperatures. It was supposed that the esterase was endowed with a habitat-specific characteristic due to low fermentation of Mao-tofu [27]. There were also several reports on cold-adapted microbial esterases/lipases that showed similar catalytic activity at such low temperatures. The optimum temperature of Est684 was slightly lower than Lp_2631 from *Lactobacillus plantarum* [28], and these cold-adapted esterases/lipases reported by Yu et al. and Mander et al. [29, 30]. In the light of cold activity, Est684 displayed slightly better performance than Est12 from *Psychrobacter celer* 3Pb1 which showed 41% activity at 0 °C [31], but worse than other cold-active esterases, such as EstLiu from *Zunongwangia profunda* which showed 75% activity at 0 °C [11], est10 from *Psychrobacter pacificensis* which showed 55% activity at 0 °C [32], and EstPc from *Psychrobacter cryohalolentis* K5T, showed 80% of activity at 0 °C [33].

Thermostability was determined by analysis of the residual activity toward *p*-nitrophenyl butyrate at regular intervals after preincubation for durations up to 24 h, at temperatures ranging from 18 to 35 °C. Est684 was very stable at 18 °C, with 52% residual activity after incubation for 18 h (Fig. 3b). However, the activity decreased dramatically at 35 °C, and remained 28% activity for 2 h, much worse than Sys410 and its mutants [34, 35], which proved that thermostability and high activity at low temperatures are apparently incompatible in natural enzymes.

### Immobilization of purified esterase onto mesoporous silica SBA-15

Successful application of a catalyst depends to a significant extent on its stability and recyclability. Immobilization of enzymes onto suitable materials is a proven strategy. In the current study, three kinds of carriers were chosen to study Est684 immobilization. In all cases, 100% of the enzyme was immobilized in a short time, moreover, the remaining activity on chitosan beads, sodium alginate and mesoporous silica SBA-15 were 10.1, 12.7 and 66.3%, respectively (Table 1). Mesoporous silica SBA-15 were therefore selected to further optimize reaction conditions.

**Table 1:** The remaining activity of the immobilizing Est684 on different carriers

Carriers	The remaining activity
Chitosan beads	10.1 ± 0.2
Sodium alginate	12.7 ± 2.1
Mesoporous silica SBA-15	66.3 ± 0.9

**Fig. 4:**
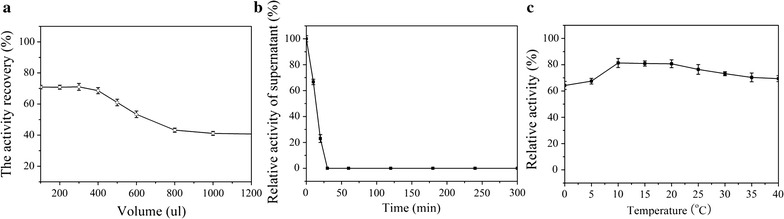
Immobilization conditions of Est684 onto mesoporous silica SBA-15. **a** The activity recovery with different enzyme amounts. **b** The relative activity of the supernatant after different reaction intervals. **c** The activity recovery at different immobilizing temperatures

Immobilization was performed with 100 mg of mesoporous silica SBA-15 with a protein concentration of 0.32 mg/mL (728 U/mL). To bind the esterase onto SBA-15, three factors were optimized: loading amount, immobilizing time, and reaction temperature. The results of these experiments are shown in Fig. 4. First, we explored the adsorption amounts of Est684 on 100 mg of mesoporous silica SBA-15 because too much enzyme would exceed the capacity of the carrier. The optimum amount of Est684 was 0.096 mg (Fig. 4a). The optimal binding time for the immobilization process was also determined (Fig. 4b). Adsorption of esterases on carriers was very rapid and complete, and nearly 100% of the enzyme were immobilized in less than 30 min, similar to a previous report [36]. After 30 min, little enzyme activity remained in the supernatant, indicating that all enzyme was immobilized on the carrier (100% immobilization). Reaction temperatures were also investigated. The results clearly showed that reaction temperature plays an insignificant role in the immobilization process (Fig. 4c). The recovery of enzyme activity was more than 60% at temperatures ranging from 0 to 40 °C. The optimal conditions were a lipase/silica ratio of 0.96 mg/g (w/w), adsorption time of 30 min, and a temperature of 10 °C. Under such conditions, the activity recovery remained at 81.3%. This report is the first about immobilizing pyrethroid-hydrolyzing enzymes on mesoporous silica.

### SEM analysis

**Fig. 5:**
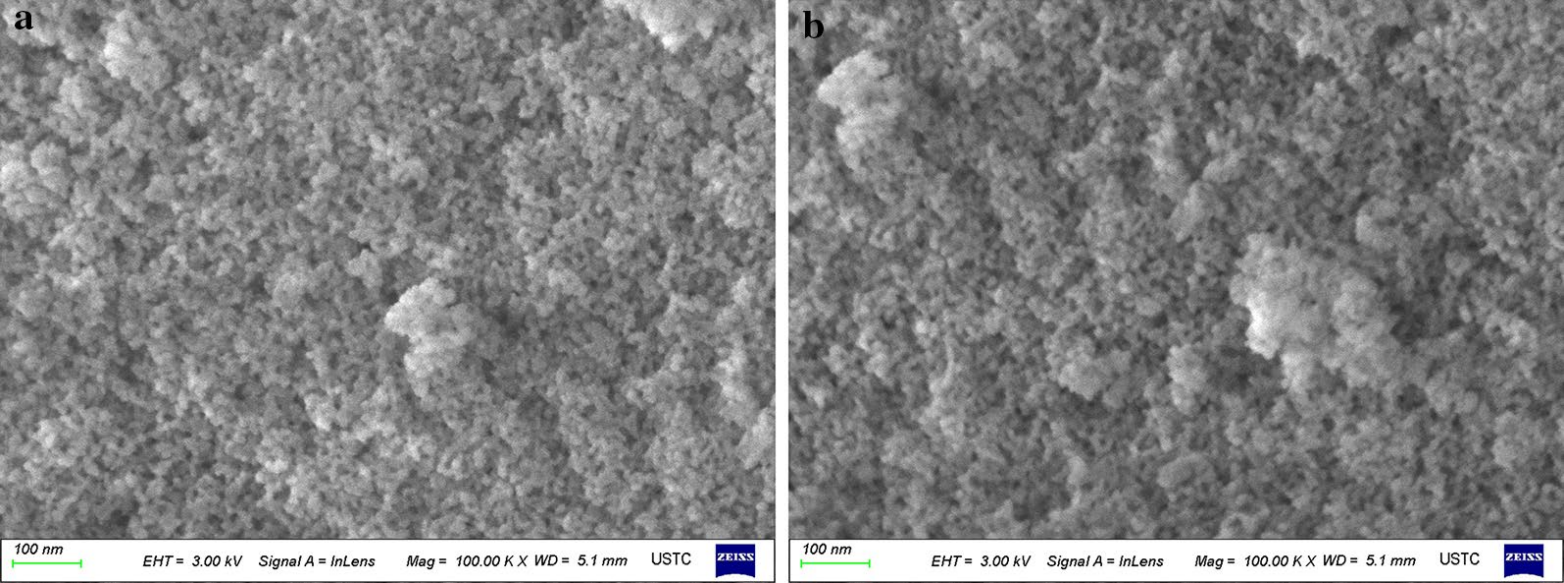
Micrographs of the morphologies of the mesoporous silica SBA-15 without (**a**) and with (**b**) bound enzymes

The morphology of the mesoporous silica SBA-15 with and without bound esterase was assessed by scanning electron microscopy with an extra high tension setting of 3.0 kV. The images in Fig. 5 show channels with a uniform size of about 10 nm in diameter, similar to those reported previously [37]. The morphology of the materials did not change after the immobilization of the enzyme, which confirmed that mesoporous silica SBA-15 is stable after enzyme immobilization.

### Kinetic parameters determination

*p*-Nitrophenyl butyrate was used to test the activity and kinetic parameters of the soluble and immobilized enzyme. The *K*m and *k*cat values were calculated by fitting the data to the Michaelis–Menten equation. The *K*m and *k*cat values of the soluble enzyme were 6.01 ± 2.74 μM and 417.15 ± 1.51 s^−1^, respectively. The *K*m and *k*cat values of the immobilized enzyme were 7.92 ± 2.10 μM and 403.47 ± 2.71 s^−1^, respectively. The *K*m value was much lower than the pyrethroid-hydrolyzing esterase Sys410 and its mutants [34, 35], which demonstrated that both soluble and immobilized Est684 had better affinity for the substrate. Compared to the soluble enzyme, *K*m of the immobilized Est684 showed a little increase upon immobilization, which reflected a slightly decreased affinity for the substrate and the presence of partitioning and diffusional effects in the pores of the SBA-15 matrix. The slight change in the affinity for *p*-nitrophenyl acetate may have been caused by structural changes in the enzyme introduced by the immobilization procedure and lower accessibility of the active site of the immobilized enzyme for the substrate [38, 39]. Nevertheless, the immobilized enzyme had high catalytic activity and a high *k*cat value. Efficient catalytic activity is a valuable enzymatic property for practical applications.

### Comparison of catalytic properties of soluble and immobilized Est684

**Fig. 6:**
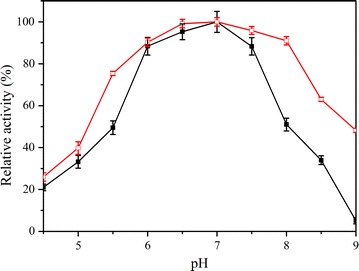
Effect of pH on the activity of soluble (filled square) and immobilized Est684 (open square). The optimum pH was measured using *p*-nitrophenyl acetate as a substrate at 18 °C. Data points are the average of triplicate measurements, and error bars represent the standard deviation

Enzyme activity can be significantly influenced by pH and temperature. In this study, the effect of pH on enzyme activity was determined between pH 4.5 and pH 9.0. The optimal pH of the immobilized enzyme was 6.5–7.0, different from that of the soluble enzyme (pH 7.0) (Fig. 6). Interestingly, the relative activity of the immobilized enzyme was more than 75% at the pH range between 5.5 and 8.0, while soluble enzyme attained activity over 50% at the same pH range. The immobilized enzyme had a higher activity than the soluble enzyme over a range of pH values. Good pH adaptability is attractive when dealing with fluctuating conditions during bioremediation.

**Fig. 7:**
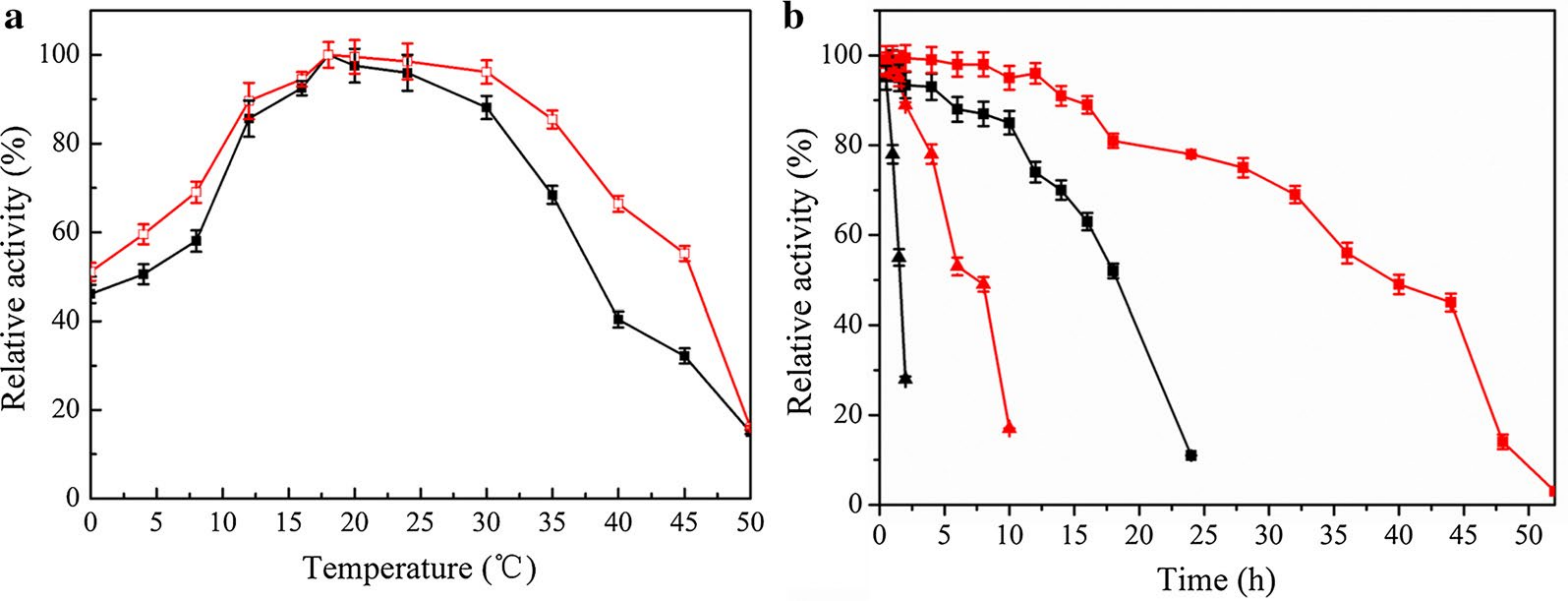
Effect of temperature on the activity and stability of the soluble and immobilized Est684. **a** The optimum temperature of the soluble (filled square) and immobilized (open square) Est684 was determined analogously by measuring esterase activity in the temperature range of 0–50 °C in a phosphate buffer (50 mM, pH 6.5). **b** Thermostability of the soluble (red line) and immobilized (black line) Est684 was measured by preincubation of enzymes in 50 mM potassium phosphate buffer (pH 6.5) at 18 (filled square) and 35 °C (filled triangle) for different intervals. Data points are the average of triplicate measurements, and error bars represent the standard deviation

The activity of the soluble and immobilized Est684 was determined at temperatures of 0–50 °C (Fig. 7a). The soluble and immobilized enzyme had the same optimal temperature of 18 °C, but the immobilized enzyme displayed more than 50% relative activity at temperatures ranging from 0 to 45 °C. This range was broader than that for the soluble enzyme, demonstrating that the immobilized pyrethroid-hydrolyzing enzyme possesses remarkable adaptability at low and moderate temperatures. Thermostability was determined by analysis of residual activity after preincubation at 18 and 35 °C for different time intervals (Fig. 7b). The half-life (T_1/2_) of the soluble enzyme at 35 °C was 1.5 h (55.1%), while the half-life (T_1/2_) of the immobilized enzyme at 35 °C was 6 h (53.8%), 4 times longer than the soluble one. The half-life (T_1/2_) at 18 °C was extended for 22 h. These results prove that mesoporous silica SBA-15 is suitable for immobilizing Est684 and significantly improves its thermostability. The immobilization matrix appears to protect the enzyme from being denatured and restricts conformational change. For practical applications, thermostability enhancement of the targeted enzyme is generally attractive because it extends the shelf life of the enzyme in reagents.

### Operational stability of the immobilized Est684

**Fig. 8:**
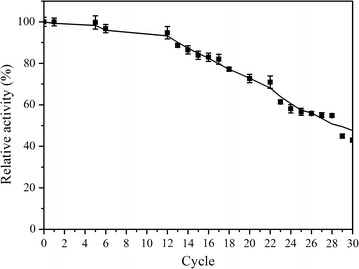
Operational stability of the immobilized Est684. The immobilized enzyme was incubated with *p*-nitrophenyl acetate for 5 min at 30 °C followed by centrifugation (4000*g*, 1 min) at 4 °C, and then repeatedly used for up to 30 batch reactions

The reusability of immobilized enzymes is very important for industrial applications. The operational stability of the immobilized Est684 on mesoporous silica SBA-15 was investigated for 30 consecutive cycles. The relative activity on reuse numbers is illustrated in Fig. 8. Little loss in enzyme activity occurred up to 12 consecutive cycles, after which a slight decrease was observed. After 28 cycles, the enzyme still retained nearly 54% of its activity. The slight decrease in enzyme activity during repeated use might be due to enzyme leakage from carriers during operation, because the bonding is relatively weak. The immobilized enzyme could apparently be reused for 28 cycles without substantial loss of activity.

The outstanding operational stability could be attributed in part to the suitable pore size of SBA-15. Supports with small pore diameters would impede the exit of enzyme, while large pore diameters offer less resistance to enzyme release [40]. The good thermostability of Est684 itself may contribute to the stability. Xu et al. [41] reported that the relative activity of NH-SBA-15-PPL remains at 49.2% after the 5th reuse. Cai et al. [42] reported that 92.5% of the initial glycerolysis activity of SBA-15(6.6)-CALB remained after five cycles, each lasting 12 h. SBA-15 clearly serves as an excellent support for immobilizing enzymes, which enhances operational stability and provides a highly biocompatible environment for enzymes. These results suggest that the immobilized Est684 possesses high catalytic activity, outstanding operational stability, and good adaptability at low and moderate temperatures, which may be useful in the bioremediation of pyrethroids. However, further experiments are needed to ensure the suitability of the procedure for removal of pesticides from vegetables.

### Gas chromatography analysis of the degradation of pyrethroids in cucumbers

**Fig. 9:**
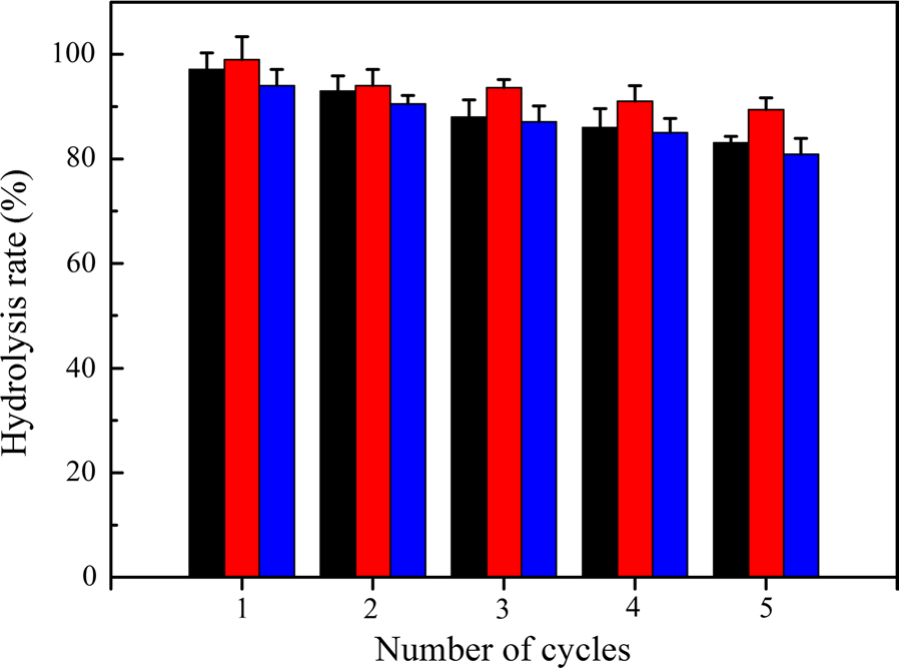
The hydrolysis rate of cyhalothrin (black), cypermethrin (red) and fenvalreate (blue) in the pyrethroid-contaminated cucumbers degrading by the immobilized Est684. The immobilized enzyme was investigated for five consecutive cycles. Data points are the average of triplicate measurements, and error bars represent the standard deviation

**Fig. 10:**
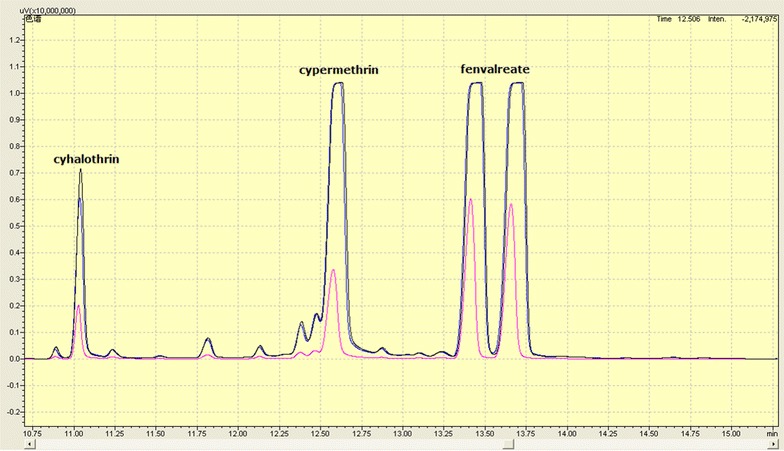
Gas chromatography analysis of different pyrethroids hydrolyzed by Est684 after 5th cycle. The black, blue, and red lines denote hydrolysis chromatography of blank group, control group, and experimental group, respectively

In the present study, organic cucumbers were used to assess the potential application of immobilized Est684 to biodegrade pyrethroid residues. Assays for pyrethroid hydrolysis were performed at 18 °C for 5 min with slow stirring for several reasons. Our aim was to evaluate the effectiveness of the enzyme at dish washing temperature, and 5 min was in accordance with the average time that people spend in washing vegetables and fruits. These conditions could simulated washing vegetables and fruits under typical conditions. The ability to hydrolyze various pyrethroids was determined by gas chromatography analysis. The results are shown in Fig. 9. The immobilized enzyme could still efficiently hydrolyze pyrethroids on contaminated cucumbers. The hydrolysis rate of three kinds of pyrethroids reached over 90% after two cycles within 5 min, and still remained more than 85% after four cycles. The results of the 5th cycle is shown in Fig. 10. In the control group, water immersion only slightly degraded these three pyrethroids with 2–10% degrading efficiency for 5 min. Interestingly, the immobilized enzyme was able to efficiently hydrolyze all three pyrethroids tested within a very short time, and the hydrolysis rates of cyhalothrin, cypermethrin and fenvalreate were 83.1, 89.4, and 80.9%, respectively. These results indicate that the immobilized enzyme retained broad substrate specificity and the high activity of the soluble enzyme.

## Conclusions

In this study, we identified a novel pyrethroid-hydrolyzing esterase Est684 from the Mao-tofu metagenome for the first time. The recombinant enzyme displayed high activity but weak stability. With the aim of improving stability and recycling enzymes, Est684 was immobilized on mesoporous silica SBA-15 using absorption method. The results showed that immobilization could improve the properties of the enzyme. Thermostability and operational stability were notably enhanced, and the immobilized enzyme retained broad substrate specificity and catalytic activity. The enzyme efficiently hydrolyzed several pyrethroids on contaminated cucumbers within a very short time, reaching over 90% degradation efficiency after two cycles. These favorable features make the process ideal for biodegradation of pyrethroids on contaminated vegetables.

## Materials and methods

### Chemicals and materials

All *p*-nitrophenyl esters were purchased from Sigma. Mesoporous silica SBA-15 (<150-μm particle size, 8-nm pore size) was purchased from Rusology Technology Co. LTD (Guangxi, China). The chitosan beads, Chitopearl BCW-3010 (BCW), were purchased from Wako Chemicals GmbH (Neuss, Germany). T4 DNA ligase, restriction endonuclease, and DNA polymerase were purchased from TaKaRa (Dalian, China) and used according to manufacturer recommendations. E.Z.N.A. Plasmid Mini Kit and E.Z.N.A. Gel Extraction Kit were purchased from OMEGA (Norcross, USA). All chemicals were of analytical or electrophoresis grade, and unless stated otherwise, they were purchased from Sigma Aldrich (St. Louis, MO, USA).

### Bacterial strains and plasmids

*Escherichia coli* DH5α and *E. coli* BL21 (DE3) (Novagen, Madison, WI, USA) was used as the host for gene cloning and protein expression. pUC118 (TaKaRa) and pET28a (+) (Novagen) was used to construct metagenomic library and express the target gene.

### Metagenomic library construction and pyrethroid-hydrolyzing gene screening

Microbes from Mao-tofu were used to construct one metagenomic library. The total DNA was extracted on the base of the method described before [26, 43]. The purified DNA was digested into 3.0–10 kb fragments by incomplete digestion of *Eco*RI. The metagenomic library was constructed based on the previous method [26]. Initially, the transformants were replicated on LB agar plates supplemented with 0.1% (v/v) tributyrin. Transparent zone due to tributyrin hydrolysis appeared after cultivation at 37 °C for 1–2 days. The positive clones with hydrolysis zones were selected, and further tested for the ability to hydrolyze pyrethroids confirmed by gas chromatography (GC) analysis. One clone with pyrethroid-hydrolyzing activity was screened. The recombinant plasmid (pUC118No.2) was sequenced on ABI 377 DNA sequencer.

### Cloning, expression, and purification of pyrethroid-hydrolyzing esterase

The putative pyrethroid-hydrolyzing gene *est684* was amplified from the pUC118No.2 plasmid by using the primers. The following primers were used: fw (5′- CGCGGATCCATGCCGTCTGGAGAAAATCAGTTTT-3′; the *Bam*HI cutting site is underlined) and rv (5′- CCCAAGCTTCTACTTCTTCAGCGTTTCCTCGAAC-3′; the *Hind*III cutting site is underlined). Amplified DNA was digested by *Bam*HI/*Hind*III, ligated into the vector pET-28a (+) which was linearized by *Bam*HI/*Eco*RI, then transformed into *E. coli* BL21 (DE3) cells. The procedure was carried out according to the method of Fan et al. [26]. Recombinant cells were cultivated in a 250-mL flask containing 50 mL of Luria–Bertani (LB) (50 μg/mL kanamycin) at 37 °C until the cell concentration reached an OD_600_ of 1.0, then induced with 0.6 mM isopropyl β-d-1-thiogalactopyranoside (IPTG) at 37 °C for 8 h with shaking at 220 rpm. The protein of interest was purified with a Ni–NTA His·Bind column according to the method of Fan et al. [34]. The sample was loaded onto a Ni–NTA His·Bind column pre-equilibrated with binding buffer. Then the column was washed with binding buffer and washing buffer (0.5 M NaCl, 60 mM imidazole, 20 mM Tris–HCl, pH 7.9). Finally, the bound protein was eluted with eluting buffer (1 M imidazole, 0.5 M NaCl, 20 mM Tris–HCl, pH 7.9). The eluted samples were further purified using a Superdex 200 (16/60) size-exclusion column (GE Healthcare) with 20 mM Tris–HCl, 200 mM NaCl, pH 7.5 [44]. The purified protein corresponding to Est684 was collected and stored at −80 °C. The molecular mass of the denatured protein was determined by sodium dodecyl sulfate–polyacrylamide gel electrophoresis (SDS-PAGE). A 12% SDS-PAGE was prepared by the previous method [45]. Proteins were stained with Coomassie brilliant blue G-250.

### Determination of substrate specificity

*p*-nitrophenyl esters, as general substrates of esterases/lipases, were used to determine substrate specificity of est684 according to the method of Fan et al. [26], with slight modification. The reaction was performed at pH 6.5 and 18 °C for 5 min. The production of ρ-nitrophenol was measured at 405 nm by Labsystems Dragon Wellscan MK3. One unit of enzyme activity was defined as the rate of the reaction that the enzyme produced 1 μmol of ρ-nitrophenol per minute under the conditions. In each measurement, the effect of nonenzymatic hydrolysis of substrates was considered and subtracted from the value measured when the enzyme was added.

### Effect of temperature on activity and stability of recombinant enzyme

The optimum temperature was evaluated by testing esterases/lipases activity at pH 6.5 in temperature ranges of 0–50 °C based on the previous method [26], with slight modification. Thermostability was measured by preincubation of the purified enzyme in 50 mM potassium phosphate buffer (pH 6.5) at different temperatures for intervals. Then, the residual activity was measured using *p*-nitrophenyl butyrate as a substrate at pH 6.5 and 18 °C based on the method described previously [26].

### Investigation of immobilization conditions

Immobilization of the pyrethroid-hydrolyzing esterase on chitosan beads was performed using glutaraldehyde as the crosslinker [46]. The immobilization on alginate was performed as previously described [47], with some modifications as follows: 4% sodium alginate, 1 (mg/g) enzyme/sodium alginate ratio, 4% CaCl_2_, and 4 h of crosslinking time at 4 °C.

Immobilization of the enzyme on mesoporous silica SBA-15 was performed as follows: the enzyme solution was diluted by potassium phosphate buffer (pH 7.5) to a specific concentration (728 U/mL, 0.32 mg/mL). One hundred milligrams of SBA-15 was dispersed in appropriate amounts of Est684. The volumes varied from 100 to 2000 μL. After 4 h of incubation at 4 °C with shaking at 100 rpm, the suspension was centrifuged (6000×*g*, 2 min) to precipitate the bound enzymes, which were then resuspended in potassium phosphate buffer. The process was repeated 3 times to ensure removal of the unbound enzyme.

The effects of varying immobilizing time and temperature for the immobilization of the esterase onto mesoporous silica SBA-15 were investigated separately. One hundred milligrams of carrier was dispersed in 300 μL of Est684 (728 U/mL). After incubation at 4 °C with shaking at 100 rpm for various intervals, the unbound enzyme was washed out and the immobilized enzyme was obtained. Immobilizing intervals were 10, 20, 30 min, 1, 2, 3, 4, and 5 h. For experiments to test the effect of the immobilizing temperature, 100 mg of carrier was dispersed in 300 μL of Est684 (728 U/mL, 0.32 mg/mL). After incubation at 0, 5, 10, 15, 20, 25, 30, 35, or 40 °C at 100 rpm for 30 min, the unbound enzyme was washed out and immobilized enzyme was obtained.

The activity of the immobilized enzyme and the supernatant was measured using *p*-nitrophenyl butyrate as a substrate at pH 6.5 and 18 °C. The immobilized enzyme was dried at room temperature and stored at 4 °C for further study. The activity recovery was defined as the activity of the immobilized enzyme compared to the total starting activity of the free enzyme: the activity recovery (%) = (Immobilized enzyme activity/total enzymes activity) × 100%.

### Material characterization

The samples were coated with platinum and analyzed using Scanning Electron Microscopy (SEM) (Hitachi SU3500, Krefeld, Germany) using the secondary electron emission mode at 1 kV. Images were collected at 100,000× magnifications.

### Determination of kinetic parameters

Enzyme activity was tested under conditions of pH 6.5 and 18 °C by using *p*-nitrophenyl butyrate as a substrate, according to the method of Fan et al. [35]. The free and immobilized enzymes were incubated with various concentrations of *p*-nitrophenyl butyrate. The final concentrations ranged from 1.0 to 10.0 mM in potassium phosphate buffer (pH 6.5). The kinetic parameters were calculated by fitting the initial rate data into the Michaelis–Menten equation using GraFit software version 6 (Erithacus Software Ltd., Horley, UK).

### Comparison of catalytic properties of free and immobilized enzyme

Effects of temperature and pH on the reaction rates of free and immobilized enzyme were determined by using *p*-nitrophenyl butyrate as a substrate. The optimum pH was measured at 18 °C. The pH buffers included citric acid-NaOH buffer (pH 3.5–5.5), potassium phosphate buffer (pH 5.0–7.0), and Tris–HCl buffer (pH 6.5–9.0). The optimum temperature and thermostability were determined according to the method 2.6.

### Operational stability of the immobilized enzyme

To evaluate the operational stability of the immobilized enzyme, the immobilized enzyme was incubated with *p*-nitrophenyl butyrate for 5 min at 18 °C followed by centrifugation (4000×*g*, 1 min) at 4 °C. The immobilized enzyme was repeatedly used to catalyze *p*-nitrophenyl butyrate for up to 30 batch reactions. The residual activity (%) was determined as follows:

$$ {\text{Residual activity }}\left( \% \right) = \frac{{{\text{enzyme activity in }}n{\text{th cyle}}}}{{{\text{enzyme activity in }}1{\text{st cyle}}}} \times 100\% $$

### Gas chromatography analysis of the degradation of different pyrethroids in cucumbers

Cucumbers are one of the most common consumed vegetables. In this study, organic cucumbers without pesticide application during growth were collected from an experimental plot of Dongguan Agriculture Research Center. Fresh cucumbers were immersed in pyrethroid solutions consisting of 0.2 mg/L cyhalothrin, 0.2 mg/L cypermethrin, and 0.2 mg/L fenvalreate for 5 min and then air-dried in a fume hood. The pyrethroid-contaminated cucumbers were randomly divided into three groups, including a blank group, control group, and experimental group. For each group, approximately 1 kg of pyrethroid-contaminated cucumbers were weighed into a plastic pot. Control samples were immersed in 1.0 L of tap water at 30 °C for 5 min with slow stirring and then air-dried in fume hood. For the experimental group, 1 kg of cucumbers were immersed in 1.0 L of tap water containing 10.0 mg of immobilized enzyme (1.5 U/mg) at 18 °C for 5 min with slow stirring and then air-dried in fume hood. The immobilized enzyme was then collected by natural precipitation and filtration, and then used for the next four reactions. Blank samples without any further processing, control samples, and experimental samples were separately blended in a variable speed laboratory blender until homogenized. Ten grams of each comminuted homogenous sample was placed a 50-mL centrifuge tube. Next, 20 mL of acetonitrile and 4.0 g of sodium chloride were added, and the samples were immediately vortexed for 20 min and then centrifuged for 5 min at 6000×*g*. A 4.0-mL aliquot of each extract was transferred into a 20-mL glass cuvette and dried under nitrogen, after which the extract was redissolved in 2 mL of n-hexane. The residues detected by the gas chromatography analysis were based on the method described previously [34]. All experiments were carried out in triplicate.

## Additional file

**Additional file 1: Figure S1.** SDS-PAGE of gene expression in *E. coli* BL21 (DE3). M, protein MW marker; lanes 1, purified Est684.
