# Supplementary figures and images for: Identification and immobilization of a novel cold-adapted esterase, and its potential for bioremediation of pyrethroid-contaminated vegetables
Source: Microb Cell Fact. 2017 Sep 11;16:149. doi: 10.1186/s12934-017-0767-9 (PMC5594479; doi:10.1186/s12934-017-0767-9)

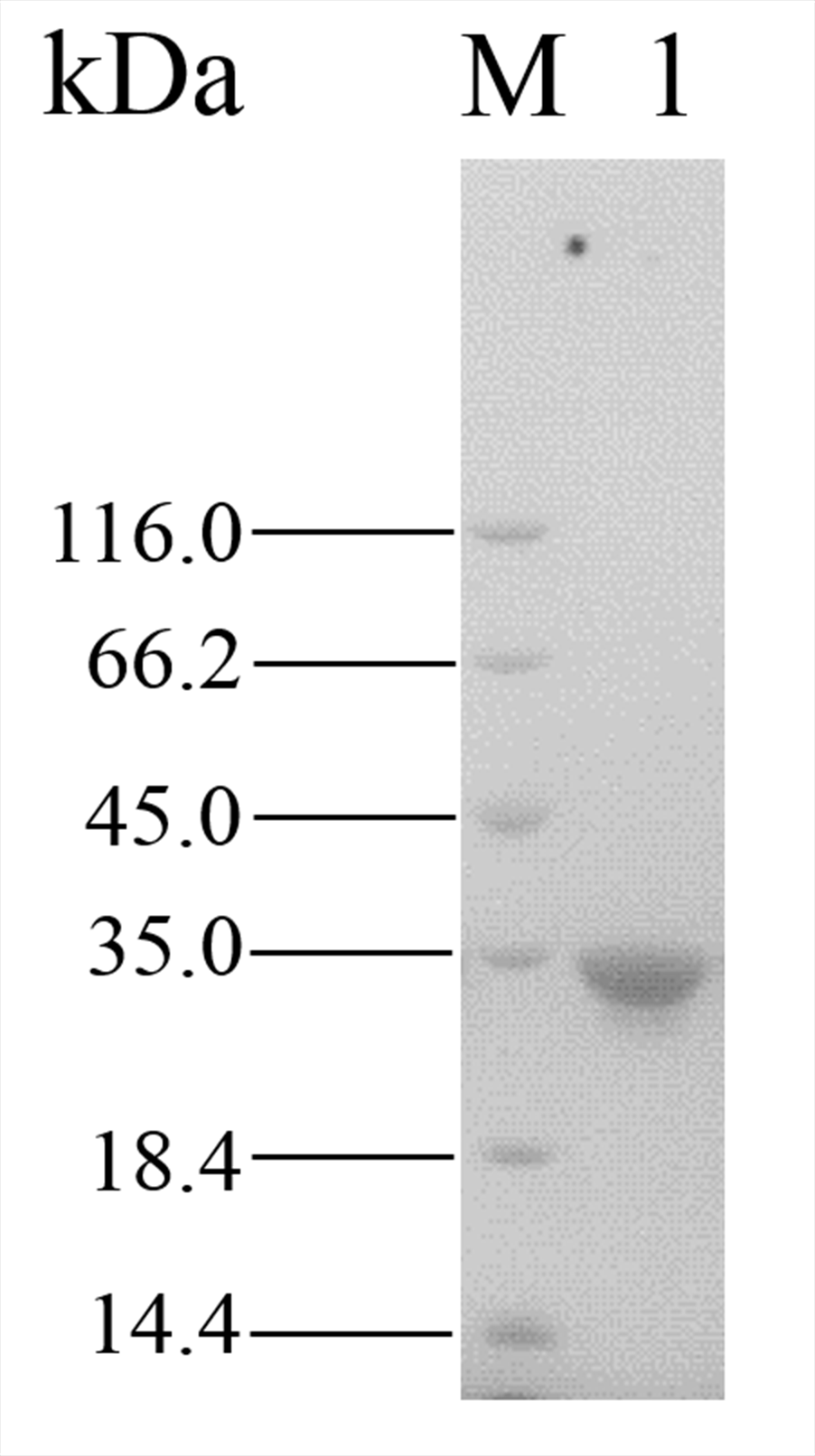

Supplement: Supplementary file 1 — Additional file 1: Figure S1. SDS-PAGE of gene expression in E. coli BL21 (DE3). M, protein MW marker; lanes 1, purified Est684. [file 12934_2017_767_MOESM1_ESM.tif]
